# Liver biomarkers, lipid metabolites, and risk of gestational diabetes mellitus in a prospective study among Chinese pregnant women

**DOI:** 10.1186/s12916-023-02818-6

**Published:** 2023-04-17

**Authors:** Ping Wu, Yi Wang, Yi Ye, Xue Yang, Yichao Huang, Yixiang Ye, Yuwei Lai, Jing Ouyang, Linjing Wu, Jianguo Xu, Jiaying Yuan, Yayi Hu, Yi-Xin Wang, Gang Liu, Da Chen, An Pan, Xiong-Fei Pan

**Affiliations:** 1 Department of Epidemiology and Biostatistics, Ministry of Education Key Laboratory of Environment and Health, School of Public Health, Tongji Medical College, Huazhong University of Science and Technology, Wuhan, 430030 Hubei China; 2 Department of Epidemiology and Biostatistics, West China School of Public Health and West China Fourth Hospital, Sichuan University, Chengdu, 610041 Sichuan China; 3 Department of Toxicology, School of Public Health, Anhui Medical University, Hefei, 230032 Anhui China; 4 Department of Clinical Laboratories, Shuangliu Maternal and Child Health Hospital, Chengdu, 610200 Sichuan China; 5 Department of Science and Education, Shuangliu Maternal and Child Health Hospital, Chengdu, 610200 Sichuan China; 6 Department of Obstetrics and Gynecology & Ministry of Education Key Laboratory of Birth Defects and Related Diseases of Women and Children, West China Second University Hospital, Sichuan University, Chengdu, 610041 Sichuan China; 7 Department of Nutrition and Food Hygiene, Hubei Key Laboratory of Food Nutrition and Safety, School of Public Health, Tongji Medical College, Huazhong University of Science and Technology, Wuhan, 430030 Hubei China; 8 School of Environment, Guangdong Key Laboratory of Environmental Pollution and Health, Jinan University, Guangzhou, 511436 Guangdong China; 9 Section of Epidemiology and Population Health & Department of Obstetrics and Gynecology, Ministry of Education Key Laboratory of Birth Defects and Related Diseases of Women and Children & National Medical Products Administration Key Laboratory for Technical Research on Drug Products In Vitro and In Vivo Correlation, West China Second University Hospital, Sichuan University, Sichuan Chengdu, 610041 China; 10 Shuangliu Institute of Women’s and Children’s Health, Shuangliu Maternal and Child Health Hospital, Chengdu, 610041 Sichuan China

**Keywords:** Hepatic steatosis index, Liver enzyme, Gestational diabetes mellitus, Lipidomics, Birth cohort

## Abstract

**Background:**

Liver plays an important role in maintaining glucose homeostasis. We aimed to examine the associations of liver enzymes and hepatic steatosis index (HSI, a reliable biomarker for non-alcoholic fatty liver disease) in early pregnancy with subsequent GDM risk, as well as the potential mediation effects of lipid metabolites on the association between HSI and GDM.

**Methods:**

In a birth cohort, liver enzymes were measured in early pregnancy (6-15 gestational weeks, mean 10) among 6,860 Chinese women. Multivariable logistic regression was performed to examine the association between liver biomarkers and risk of GDM. Pearson partial correlation and least absolute shrinkage and selection operator (LASSO) regression were conducted to identify lipid metabolites that were significantly associated with HSI in a subset of 948 women. Mediation analyses were performed to estimate the mediating roles of lipid metabolites on the association of HSI with GDM.

**Results:**

Liver enzymes and HSI were associated with higher risks of GDM after adjustment for potential confounders, with ORs ranging from 1.42 to 2.24 for extreme-quartile comparisons (false discovery rate-adjusted *P*-trend ≤0.005). On the natural log scale, each SD increment of alanine aminotransferase, aspartate aminotransferase, gamma-glutamyl transferase, alkaline phosphatase, and HSI was associated with a 1.15-fold (95% CI: 1.05, 1.26), 1.10-fold (1.01, 1.20), 1.21-fold (1.10, 1.32), 1.15-fold (1.04, 1.27), and 1.33-fold (1.18, 1.51) increased risk of GDM, respectively. Pearson partial correlation and LASSO regression identified 15 specific lipid metabolites in relation to HSI. Up to 52.6% of the association between HSI and GDM risk was attributed to the indirect effect of the HSI-related lipid score composed of lipid metabolites predominantly from phospholipids (e.g., lysophosphatidylcholine and ceramides) and triacylglycerol.

**Conclusions:**

Elevated liver enzymes and HSI in early pregnancy, even within a normal range, were associated with higher risks of GDM among Chinese pregnant women. The association of HSI with GDM was largely mediated by altered lipid metabolism.

**Supplementary Information:**

The online version contains supplementary material available at 10.1186/s12916-023-02818-6.

## Background

Gestational diabetes mellitus (GDM) is one of the most common complications in which glucose intolerance develops during pregnancy and begets short- and long-term major adverse health consequences for both mothers and the offspring [1]. The prevalence of GDM varied globally, ranging from 1% to >30% [1]. In China, the average prevalence of GDM was reported to be about 14.8%, with wide variations across regions [2]. However, the pathophysiology of GDM is complex and remains to be delineated. Exploring early biomarkers for GDM and further understanding their roles in the onset of GDM is of substantial importance.

Non-alcoholic fatty liver disease (NAFLD), which involves insulin resistance in its development [3], was reported to predict dysglycemia in mid-pregnancy [4, 5]. However, it is impractical to widely perform ultrasound screening for NAFLD or liver biopsy in asymptomatic pregnant women. Hepatic steatosis index (HSI), indicated as a reliable noninvasive marker for NAFLD [6, 7], could have an agreement of 75.3% with transient elastography in the Asian population [7]. Evidence emerges that it could have a potential to predict women with a higher risk of subsequent GDM [5, 8]. Lipidomics is an useful tool to understand the etiology of metabolic diseases [9]. Several NAFLD-associated lipid metabolites have been reported, such as lysophosphatidylcholine (LPC) [10–13], sphingomyelin (SM) [12, 14], ceramides (Cer) [15, 16], and triacylglycerol (TG) [10, 12–14, 17]. Of note, lipid metabolites have also been reported to be involved in the development of GDM [18, 19]. It is unclear whether certain lipid metabolites may mediate the association between HSI and risk of GDM.

In addition, elevated liver enzymes are commonly observed in NAFLD [20] and may be involved in insulin resistance [21]. A recent meta-analysis of eight studies reported that gamma-glutamyl transferase (GGT) was a significant and robust predictor of incident GDM in pregnant women [22], while the association between alanine aminotransferase (ALT) levels in early pregnancy and GDM risk was inconsistent [23–25] and studies on alkaline phosphatase (ALP) or aspartate aminotransferase (AST) were scarce [22]. Thus, the associations between liver enzymes in early pregnancy and GDM are still inconsistent.

Therefore, our primary aim was to investigate the association between HSI in early pregnancy and subsequent GDM risk and further assess whether this association was mediated by lipid metabolites in a Chinese birth cohort. In addition, we secondarily examined the associations between different liver enzymes in early pregnancy and risk of GDM.

## Methods

### Study design and participants

The Tongji-Shuangliu Birth Cohort is a prospective study launched in 2017. Singleton pregnant women aged 18-40 years were invited to participate in the study at 6-15 weeks of gestation. Women were excluded if they: 1) had infertility treatment; 2) had severe chronic or infectious diseases (e.g., cancer, hepatitis B, tuberculosis, or HIV infection); or 3) were unable to or refused to complete the questionnaire. Participants provided written informed consent at enrollment. All participants were invited to complete surveys and laboratory testing at or near the time of enrollment. Blood samples were collected at baseline after 12 h overnight fasting. The cohort was approved by the Ethics Committee of the Tongji Medical College, Huazhong University of Science and Technology (Wuhan, China).

Until 2020, a total of 7,281 eligible pregnant women were included. We further excluded participants who: 1) did not have liver function testing in early pregnancy (*n*=166); 2) did not have diagnosis information of GDM (*n*=244); or 3) reported pre-pregnancy diabetes or fasting blood glucose ≥7 mmol/L in early pregnancy (*n*=11). Thus, 6,860 participants were included in our main analyses.

In the same cohort, we analyzed data from a nested case-control study of lipidomics to understand the mediating roles of lipid metabolites in association analyses. In the original nested case-control study, 336 incident GDM cases were matched to 672 controls at a 1:2 ratio on maternal age (±3 years) and gestational age (±4 weeks) [18]. After exclusion of those without liver enzymes data, we included 316 GDM cases and 632 matched controls. The study flowchart is shown in Additional file 1: Fig. S1.

### Exposure and other laboratory assessment

Serum liver enzymes (ALT, AST, GGT, and ALP) were measured for standard processing by Hitachi 7180 automatic biochemical analyzer using commercial kits (Sichuan Maccura Biotechnology, Chengdu, China) in the hospital’s clinical laboratory at or close to enrollment. The inter- and intra-assay coefficients of variation were less than 10%. Meanwhile, fasting blood glucose was measured by a commercial glucose oxidase kit (Sichuan Maccura Biotechnology, Chengdu, China). In the nested case-control study, high‐density lipoprotein cholesterol (HDL‐C), low‐density lipoprotein cholesterol (LDL‐C), total cholesterol, and triglycerides were analyzed by the BS‐200 automatic biochemistry analyzer using commercial kits (Mindray Medical International, Shenzhen, China). Serum C-reactive protein (CRP) was quantified with the Human Quantikine ELISA kits (R&D Systems, Minneapolis, MN, USA). Glycated hemoglobin (HbA1c) was analyzed by Siemens DCA Vantage HbA1c analyzer (Siemens, Berlin, Germany). Fasting insulin and C-peptide were detected by Meso Scale Discovery (MSD) U-PLEX Platform (MSD, Rockville, MD, US). Homeostasis model assessment for insulin resistance (HOMA-IR) was calculated as fasting glucose (mmol/L)×fasting insulin (mIU/L)/22.5 [26]. We calculated HSI according to the following equation: HSI =8 × ALT [U/L]/ AST [U/L] + pre-pregnancy BMI (kg/m^2^) + 2 (female) [6]. All the above mentioned laboratory assessments used fasting blood samples collected at or close to enrollment.

### Lipidomics analyses

Plasma lipidomics analyses were performed for 948 plasma samples collected at baseline using a single-phase extraction on an ultrahigh-performance liquid chromatography-tandem mass spectrometry (UHPLC-MS) platform [27]. A total of 366 lipid metabolites from 24 lipid classes/subclasses were detected. After excluding analytes with detection rates <80% or or inter- or intra-assay coefficients of variation >20% (*n*=38), a total of 328 lipid metabolites from 21 lipid classes/subclasses were identified. More details on sample collection and lipidomic measurements were described in our previous study [18].

### Assessment of covariates

Information regarding demographics, reproductive factors, parental diabetes history, personal medical history, and lifestyle (smoking status, alcohol consumption, and physical activity) was collected at baseline using structured questionnaires. Pre-pregnancy BMI was calculated by self-reported pre-pregnancy weight (kilograms) divided by the square of height (meters). Blood pressures were measured twice by Omron electronic sphygmomanometer (Omron Healthcare, Kyoto, Japan) and averaged for analyses.

### Outcome ascertainment

Participants were screened for GDM on site using the 2-h 75 g oral glucose tolerance test (OGTT) at 24 to 28 weeks of gestation after overnight fasting, or ascertained according to diagnoses in medical records. GDM was diagnosed according to the International Association of the Diabetes and Pregnancy Study Groups criteria if they met at least one of the following thresholds: fasting blood glucose ≥5.1 mmol/L, 1-hour blood glucose ≥10.0 mmol/L, or 2-hour blood glucose ≥8.5 mmol/L [28].

### Statistical analyses

Baseline characteristics of participants were presented according to the GDM status. Categorical variables were presented as numbers (percentage), and comparisons between groups were conducted by the Chi-square test. Continuous variables were summarized as median (interquartile range, IQR), and comparisons were performed by the Wilcoxon rank-sum test. Odds ratios (ORs) with 95% confidence intervals (CIs) were generated in multivariable logistic regression models to determine the associations between liver biomarkers and GDM risk after adjustment for maternal age (continuous), gestational age (continuous), parity (0 and ≥1), family history of diabetes (yes and no), history of GDM (yes and no), pre-pregnancy BMI (continuous), systolic blood pressure (continuous), smoking status (current/former and never), alcohol consumption status (current/former and never), physical activity (continuous), and fasting blood glucose (continuous). Given that pre-pregnancy BMI was included to compute HSI, pre-pregnancy BMI was treated as a categorical variable (<18.5, 18.5-24.0, and ≥24.0 kg/m^2^ according to the criteria in China [29]) in the corresponding analyses with HSI as the exposure to avoid multicollinearity. In all models, liver biomarkers were modelled as categorical variables (quartiles of original levels) and continuous variables (per SD increments after natural log-transformation to approximate a normal distribution). We tested the linear trend of the association by assigning the median value of each quartile as a continuous variable to the model. False discovery rate (FDR) correction, using the Benjamini–Hochberg procedure, was applied to adjust for multiple testing [30]. In order to examine whether there was a threshold for the dose-response relationship, restricted cubic splines were applied to assess potential nonlinear associations, with 3 knots at 10 (reference), 50, and 90 percentiles after truncating values beyond the first and 99th percentiles to avoid influence from extreme values. In addition, we estimated associations between liver biomarkers and glucose levels (fasting glucose, 1-h, and 2-h postprandial glucose levels) during OGTT. By dichotomizing liver enzymes according to their median values, the population was divided into 5 groups (0-4) based on the number of four elevated liver enzymes. We estimated the risk of GDM for participants with 1 to 4 elevated liver enzymes compared to participants with low levels of all four liver enzymes. Due to the important role of obesity and alcohol drinking in the development of fatty liver, we explored the potential effect modification (interactions) of pre-pregnancy BMI (<24 vs. ≥24 kg/m^2^ according to the criteria in China [29]) and alcohol drinking (never vs. current or former) for the association of HSI with GDM via the likelihood ratio test comparing models with and without the interaction terms.

Sensitivity analyses were done by 1) excluding participants with a history of GDM, current smokers, or current drinkers; 2) limiting participants to a relatively healthy range of liver biomarkers based on the instruction of reagent kits or previous studies (ALT ≤40 U/L, AST ≤40 U/L, GGT ≤50 U/L, ALP range from 40 U/L to 150 U/L, HSI ≤36) [5]; 3) imputing all missing values through multiple imputations by chained equations (MICE) with 10 imputations (the "multiple imputation, then deletion" approach was used, for which observations with imputed outcomes were excluded from the analysis) [31, 32]; 4) using Poisson regression model with robust error variance to estimate risk ratios (RRs) of GDM with early-pregnancy liver enzymes and HSI. Among the 6,860 participants, participants with data on metabolic markers (e.g., CRP, blood lipids, fasting insulin, and HOMA-IR) were included in Spearman partial correlation analyses to estimate the relationships of liver enzymes and HSI with metabolic traits in participants with or without GDM after adjustment for maternal age and gestational age. In the subset of 948 participants with matched pairs, we also estimated whether the associations of liver biomarkers with GDM risk were independent after further adjustment for clinical lipids, HOMA-IR, and CRP.

In the nested case-control analyses, all lipid metabolites were natural log-transformed to better approximate normal distributions and standardized to z-scores with a mean of 0 and a standard deviation (SD) of 1 before statistical analyses. Pearson partial correlation was used to assess the correlations between HSI and lipid metabolites on the log scale after adjustment for maternal age, gestational age, and GDM status. FDR correction was applied to adjust for multiple testing [30]. Lipid metabolites with the absolute value of correlation coefficients ≥0.15 and FDR-adjusted *P* <0.05 were selected for subsequent analyses. We used the least absolute shrinkage and selection operator (LASSO) regression for feature selection (“glmnet” R package) [33] and retained a parsimonious model that was most representative of HSI-related lipid metabolites. The LASSO regression model with 10-fold cross-validation was repeated 1000 times, and lipid metabolites with 100% repeatability were identified as HSI-related lipid metabolites after adjustment for maternal age, gestational age, parity, pre-pregnancy BMI, systolic blood pressure, smoking status, alcohol drinking status, physical activity, fasting blood glucose, and GDM status. A HSI-related lipid score was created by summing each LASSO-selected lipid weighted by corresponding regression coefficient. Conditional logistic regression was used to estimate the associations of HSI-related lipid metabolites or lipid score with risk of GDM after adjustment for covariates in accordance with the main analysis. We conducted mediation analyses to evaluate to what extent HSI-related lipid score may explain the association between HSI and GDM risk by using the PARAMED module in  Stata  version 15.0 (StataCorp, College Station, TX, USA). For lipid metabolites that were consistently associated with GDM risk and HSI in the same direction, we assessed the mediating role of individual lipid metabolites on the association to explore the potential mechanism through specific lipid metabolites. The mediated proportion was computed according to the formula: (indirection effect / total effect on the log scale) × 100%. To confirm the reliability of the results, another non-invasive steatosis indices, fatty liver index (FLI) [34] was also used to evaluate the association with risk of GDM in the subset population.

Analyses were performed with Stata 15.0 and R version 4.0.5 (R Foundation for Statistical Computing, Vienna, Austria). A two-tailed *P* value <0.05 was considered statistically significant unless otherwise stated (e.g., FDR-adjusted *P*-value).

## Results

### Characteristics of the participants

Of the 6,860 participants, 492 developed GDM (7.2%). Characteristics of the cohort are summarized in Table 1. The median (IQR) maternal age and gestational age in the cohort were 26 (24-29) years and 10 (9-12) weeks, respectively. Pregnant women with GDM were more likely to be older and have a history of GDM, family history of diabetes, and unfavorable metabolic traits (pre-pregnancy BMI, systolic blood pressure, and fasting blood glucose at enrollment). Similarly, women who developed GDM had significantly higher levels of liver biomarkers (ALT, AST, GGT, ALP, and HSI; all *P* <0.001).

**Table 1:** Characteristics of participants by GDM status

Characteristic	Overall (*n*=6860)	GDM (*n*=492)	No-GDM (*n*=6368)	*P* value ^a^
Age, years	26.0 (24.0-29.0)	28.0 (25.0-31.0)	26.0 (24.0-29.0)	<.001
Gestational age, weeks	10.0 (9.0-12.0)	10.0 (9.0-12.0)	10.0 (9.0-12.0)	0.76
Parity, n (%)				0.06
0	3802 (55.4)	253 (51.4)	3549 (55.7)	
≥1	3058 (44.6)	239 (48.6)	2819 (44.3)	
Family history of diabetes, n (%)	347 (5.1)	50 (10.2)	297 (4.7)	<.001
History of GDM, n (%)				<.001
Yes ^b^	82 (1.2)	34 (6.9)	48 (0.8)	
No	6778 (98.8)	458 (93.1)	6320 (99.3)	
Tobacco use, n (%)				0.15
Current or Former	464 (6.8)	41 (8.3)	423 (6.6)	
Never	6396 (93.2)	451 (91.7)	5945 (93.4)	
Alcohol use, n (%) ^c^				0.61
Current or Former	1417 (20.7)	106 (21.5)	1311 (20.6)	
Never	5443 (79.3)	386 (78.5)	5057 (79.4)	
Total physical activity, MET-h/week	121.2 (74.1-175.4)	116.3 (67.0-171.0)	121.5 (74.4-175.6)	0.07
Pre-pregnancy BMI, kg/m^2^	20.5 (19.0-22.5)	21.8 (19.9-24.1)	20.4 (18.9-22.3)	<.001
Systolic blood pressure, mmHg	108.0 (102.0-114.5)	110.5 (105.0-118.5)	108.0 (102.0-114.0)	<.001
Fasting plasma glucose, mmol/L	4.4 (4.1-4.6)	4.5 (4.2-4.9)	4.4 (4.1-4.6)	<.001
OGTT at second trimester^d^				
Fasting glucose, mmol/L	3.9 (3.7-4.2)	4.3 (4.0-4.7)	3.9 (3.7-4.1)	<.001
1-hour glucose, mmol/L	6.8 (5.7-7.9)	10.1 (9.1-10.7)	6.6 (5.6-7.6)	<.001
2-hour glucose, mmol/L	5.9 (5.2-6.8)	8.6 (7.5-9.3)	5.8 (5.1-6.6)	<.001
ALT, U/L	16.0 (12.0-24.0)	19.0 (14.0-29.0)	16.0 (12.0-24.0)	<.001
AST, U/L	18.0 (16.0-22.0)	19.0 (16.0-23.0)	18.0 (16.0-22.0)	<.001
GGT, U/L	13.0 (10.0-19.0)	16.0 (12.0-24.0)	13.0 (10.0-18.0)	<.001
ALP, U/L	46.0 (40.0-54.0)	49.0 (41.0-58.0)	46.0 (40.0-54.0)	<.001
HSI	30.0 (27.5-33.5)	32.3 (29.0-36.0)	29.9 (27.4-33.2)	<.001
HSI >36, n (%)	952 (13.9)	123 (25.0)	829 (13.0)	<.001

Data are shown as median (interquartile range) for continuous variables, and n (%) for categorical variables

^a^*P* values were calculated by Wilcoxon rank-sum test for continuous variables and Chi-square test for categorical variables. ^b^ Of 3058 women who were multiparous, 2.6% (78/3058) had a history of GDM: 14.2% (34/239) in the GDM group and 1.6% (44/2819) in the non-GDM group. ^c^ We combined the current and former drinkers because only 21 participants were current drinkers. ^d^ A total of 275, 322, and 324 participants lacked data on OGTT fasting glucose, 1-hour glucose, and 2-hour glucose during 24-28 weeks of pregnancy, respectively

*ALP* Alkaline phosphatase, *ALT* Alanine transaminase, *AST* Aspartate aminotransferase, *BMI* Body mass index, *GDM* Gestational diabetes mellitus, *GGT* Gamma-glutamyltransferase, *HSI* Hepatic steatosis index, *OGTT* Oral glucose tolerance test

### Liver biomarkers and risk of GDM

Associations of liver enzymes and HSI in early pregnancy with subsequent risk of GDM are presented in Table 2. The risk of GDM increased significantly across quartiles of all liver biomarkers, and ORs (95% CIs) for extreme-quartile comparisons ranged from 1.42 to 2.24 (FDR-adjusted *P*-trend ≤0.005; Table 2). Each SD increment of ALT, AST, GGT, ALP, and HSI on the log scale was associated with a 1.15-fold (95% CI: 1.05, 1.26), 1.10-fold (1.01, 1.20), 1.21-fold (1.10, 1.32), 1.15-fold (1.04, 1.27), and 1.33-fold (1.18, 1.51) increased risk of GDM in the fully-adjusted models, respectively. All liver biomarkers were significantly associated with higher fasting, 1-hour, and 2-hour glucose during OGTT (FDR-adjusted *P* ≤0.008; Additional file 1: Table S1). Regression based on restricted cubic splines showed nonlinear associations of ALT and AST with GDM risk (*P*-nonlinearity=0.003 and 0.01, respectively; Additional file 1: Fig. S2). In joint analyses, participants with elevated liver enzymes also showed increased risks of GDM. Compared to their counterparts, ORs for participants with 1 to 4 elevated liver enzymes were 1.13, 1.39, 1.63, and 1.95, respectively (Additional file 1: Table S2). No significant interactions were found of pre-pregnancy BMI and alcohol drinking status with HSI for GDM risk (Additional file 1: Table S3). After excluding 217 participants with a history of GDM (*n*=82), current smokers (*n*=118), or current drinkers (*n*= 21), or limiting analyses to a relatively normal range of liver biomarkers, or imputing missing values by MICE, or using Poisson regression model to estimate RRs (95% CIs) of GDM, elevated liver biomarkers were all significantly associated with increased risks of GDM (Additional file 1: Table S4-S7). In the subset population of 948 pregnant women with lipidomic data, 921 individuals had data on CRP and HOMA-IR. The positive associations of ALT, AST, and HSI with GDM were not materially changed after further adjustment for clinical lipids, CRP, and HOMA-IR, while the positive association between FLI and GDM risk became non-significant after additional adjustment for HOMA-IR (Additional file 1: Table S8).

**Table 2:** Odds ratios and 95% confidence intervals for the associations of liver biomarkers in early pregnancy with risk of GDM

Variables		Quartiles of liver biomarkers			*P*-trend ^a^	Per SD increment on the log scale	*P* for SD increment analysis ^a^
	Quartile 1	Quartile 2	Quartile 3	Quartile 4			
ALT, U/L							
Case/total (%)	92/2016 (4.6)	94/1487 (6.3)	135/1690 (8.0)	171/1667 (10.3)			
Crude model	1.00 (reference)	1.41 (1.05, 1.90)	1.82 (1.38, 2.39)	2.39 (1.84, 3.11)	<.001	1.30 (1.19, 1.41)	<.001
Model 1	1.00 (reference)	1.29 (0.95, 1.75)	1.60 (1.21, 2.12)	1.79 (1.36, 2.36)	<.001	1.17 (1.07, 1.28)	0.001
Model 2	1.00 (reference)	1.25 (0.92, 1.70)	1.54 (1.16, 2.04)	1.71 (1.29, 2.26)	<.001	1.15 (1.05, 1.26)	0.006
AST, U/L							
Case/total (%)	125/2324 (5.4)	101/1379 (7.3)	129/1643 (7.9)	137/1514 (9.1)			
Crude model	1.00 (reference)	1.39 (1.06, 1.82)	1.50 (1.16, 1.93)	1.75 (1.36, 2.25)	<.001	1.16 (1.07, 1.27)	<.001
Model 1	1.00 (reference)	1.44 (1.09, 1.90)	1.48 (1.14, 1.93)	1.55 (1.19, 2.02)	0.005	1.10 (1.01, 1.20)	0.04
Model 2	1.00 (reference)	1.46 (1.10, 1.93)	1.50 (1.15, 1.96)	1.56 (1.19, 2.04)	0.005	1.10 (1.01, 1.20)	0.04
GGT, U/L							
Case/total (%)	96/1779 (5.4)	90/1746 (5.2)	133/1754 (7.6)	173/1581 (10.9)			
Crude model	1.00 (reference)	0.95 (0.71, 1.28)	1.44 (1.10, 1.89)	2.15 (1.66, 2.79)	<.001	1.39 (1.28, 1.51)	<.001
Model 1	1.00 (reference)	0.89 (0.66, 1.20)	1.27 (0.96, 1.68)	1.64 (1.24, 2.16)	<.001	1.25 (1.14, 1.37)	<.001
Model 2	1.00 (reference)	0.85 (0.62, 1.15)	1.26 (0.95, 1.67)	1.53 (1.15, 2.03)	<.001	1.21 (1.10, 1.32)	<.001
ALP, U/L							
Case/total (%)	110/1884 (5.8)	94/1563 (6.0)	131/1726 (7.6)	157/1687 (9.3)			
Crude model	1.00 (reference)	1.03 (0.78, 1.37)	1.32 (1.02, 1.72)	1.65 (1.28, 2.13)	<.001	1.22 (1.11, 1.33)	<.001
Model 1	1.00 (reference)	1.01 (0.75, 1.35)	1.30 (0.99, 1.70)	1.51 (1.16, 1.98)	<.001	1.18 (1.07, 1.30)	0.001
Model 2	1.00 (reference)	0.98 (0.73, 1.31)	1.22 (0.93, 1.60)	1.42 (1.09, 1.86)	0.004	1.15 (1.04, 1.27)	0.007
HSI ^b^							
Case/total (%)	66/1715 (3.9)	94/1715 (5.5)	124/1715 (7.2)	208/1715 (12.1)			
Crude model	1.00 (reference)	1.45 (1.05, 2.00)	1.95 (1.43, 2.65)	3.45 (2.59, 4.59)	<.001	1.54 (1.42, 1.68)	<.001
Model 1	1.00 (reference)	1.36 (0.96, 1.93)	1.74 (1.23, 2.47)	2.51 (1.74, 3.63)	<.001	1.41 (1.25, 1.60)	<.001
Model 2	1.00 (reference)	1.31 (0.92, 1.86)	1.63 (1.14, 2.33)	2.24 (1.54, 3.25)	<.001	1.33 (1.18, 1.51)	<.001

Model 1 was adjusted for maternal age, gestational age, parity, family history of diabetes, history of GDM, pre-pregnancy BMI, smoking status, alcohol drinking status, and physical activity. Model 2 was additionally adjusted for systolic blood pressure and fasting blood glucose based on Model 1. ^a^ *P* values were corrected for multiple testing for each stepwise modeling using Benjamini–Hochberg false discovery rate method. ^b^ Pre-pregnancy BMI was treated as a categorical variable (<18.5, 18.5-24.0, and ≥24.0 kg/m^2^) for adjustment in the modeling to avoid multicollinearity

*ALP* Alkaline phosphatase, *ALT* Alanine transaminase, *AST* Aspartate aminotransferase, *BMI* Body mass index, *GDM* Gestational diabetes mellitus, *GGT* Gamma-glutamyltransferase, *HSI* Hepatic steatosis index, *SD* Standard deviation

### Liver biomarkers and metabolic profiles

Spearman partial correlation analyses included 974 participants who had data of CRP, fasting insulin, and HOMA-IR (Additional file 1: Fig. S3). ALT, GGT, ALP, and HSI, but not AST were generally associated with metabolic disturbance, such as systolic blood pressure, fasting insulin, C-peptide, HOMA-IR, CRP, and clinical lipid biomarkers among participants without GDM. GGT, ALP, and HSI were generally associated with these unfavorable metabolic markers among participants with GDM.

### HSI, lipid metabolites, and risk of GDM

**Fig. 1:**
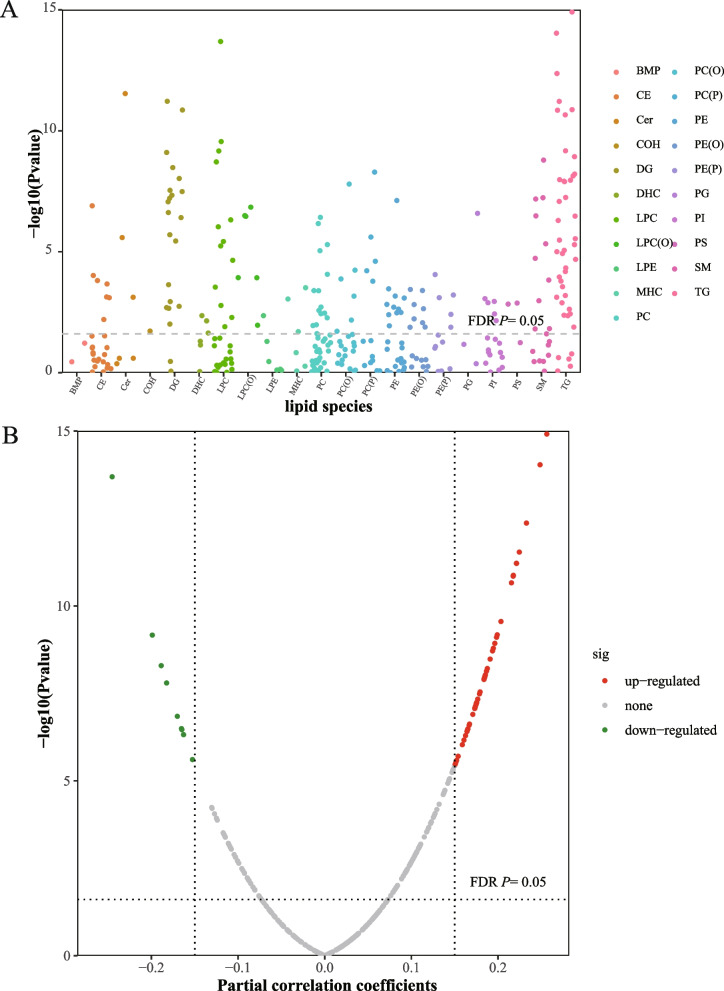
Pearson partial correlations of 328 lipid metabolites with HSI in Manhattan plot and volcano plot (*n*=948). **A** Manhattan plot showed associations of HSI with 328 lipid metabolites according to lipid classes/subclasses. **B** Volcano plot showed associations of HSI with lipid metabolites according to the significance and partial correlation coefficients. The horizontal dotted line represented the significance threshold (FDR-adjusted *P* =0.05). The scatter denoted the up-regulated (red) or down-regulated (green) lipids for correlations with HSI. Partial correlations were adjusted for maternal age, gestational age, and GDM status. Abbreviations: GDM, gestational diabetes mellitus; HSI, hepatic steatosis index; BMP, Bis(monoacylglycerol)phosphate; CE, Cholesterol ester; Cer, Ceramide; COH, free cholesterol; DG, Diacylglyceride; DHC, Dihexosyl ceramide; FDR, false discovery rate; LPC, Lysophosphatidylcholine; LPC(O), Lyso alkylphosphatidylcholine; LPE, Lyso phosphatidylethanolamine; MHC, Mono hexosyl ceramide; PC, Phosphatidylcholine; PC(O), Alkylphosphatidylcholine; PC(P), Phosphatidylcholine plasmalogen; PE, Phosphatidylethanolamine; PE(O), Alkylphosphatidylethanolamine; PE(P), Phosphatidylethanolamine plasmalogen; PG, Phosphatidylglycerol; PI, Phosphatidylinositol; PS, Phosphatidylserine; SM, Sphingomyelin; TG, Triacylglycerol

**Fig. 2:**
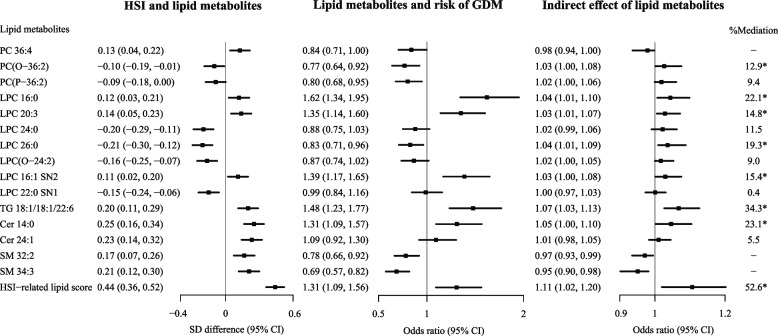
Forest plots for associations between HSI, lipids, and GDM risk (*n*=948). Results were expressed as difference (95% CI) of lipid z-score for per SD increment of HSI on the log scale and odds ratio (95% CI) of GDM for per unit increment of lipid z-score. Models were adjusted for maternal age, gestational age, parity, family history of diabetes, history of GDM, pre-pregnancy BMI, systolic blood pressure, smoke status, alcohol drinking status, physical activity, and fasting blood glucose. Pre-pregnancy BMI was treated as a categorical variable (<18.5, 18.5-24.0, and ≥24.0 kg/m^2^) in the multivariable model for associations between HSI and HSI-related lipids. Proportions of mediation were calculated according to the formula: (indirect effect / total effect on the log scale) × 100%. * Indirect effects were significant according to confidence intervals obtained by bootstrapping approach with 1000 samples. Abbreviations: BMI, body mass index; Cer, ceramide; CI, confidence interval; GDM, gestational diabetes mellitus; HSI, hepatic steatosis index; LPC, lysophosphatidylcholine; LPC(O), lyso alkylphosphatidylcholine; PC, Phosphatidylcholine; PC(O), alkylphosphatidylcholine; PC(P), Phosphatidylcholine plasmalogen; SD, standard deviation; SM, sphingomyelin; TG, triacylglycerol

Pearson partial correlation analyses showed that 59 of the 328 lipid metabolites were significantly correlated with HSI in early pregnancy, which predominantly belonged to glycerolipids, LPC, and sphingolipids (SM and Cer) (|r| ≥0.15 and FDR-adjusted *P*<0.05; Fig. 1). Among them, 49 lipid metabolites showed positive correlations with HSI (ranged from 0.150 to 0.256), while the other 10 lipid metabolites showed negative correlations (ranged from -0.260 to -0.153). The LASSO penalized generalized linear model identified 15 lipid metabolites associated with HSI after multivariable adjustments. Figure 2 showed positive associations of HSI with 9 lipid metabolites (phosphatidylcholine [PC] 36:4, LPC 16:0, LPC 20:3, LPC 16:1 stereospecific numbering 2 [SN2], TG 18:1/18:1/22:6, Cer 14:0, Cer 24:1, SM 32:2, and SM 34:3) and negative associations with 6 lipid metabolites, including alkylphosphatidylcholine (PC[O-36:2]), phosphatidylcholine plasmalogen (PC[P-36:2]), LPC 24:0, LPC 26:0, LPC 22:0 SN1, and lysoalkylphosphatidylcholine (LPC[O-24:2]). Each SD increment of natural log-transformed HSI was associated with a 0.44 (95% CI: 0.36, 0.52) SD difference of HSI-related lipid score. Of the 15 HSI-related lipid metabolites, 5 lipid metabolites were significantly associated with increased risks of GDM (LPC 16:0, LPC 20:3, LPC 16:1 SN2, TG 18:1/18:1/22:6, and Cer 14:0), whereas 6 were associated with decreased risks (PC 36:4, PC[O-36:2], PC[P-36:2], LPC 26:0, SM 32:2, and SM 34:3; Fig. 2). The HSI-related lipid score comprising the 15 identified lipid metabolites was positively associated with GDM risk, with an OR of 2.05 (95% CI: 1.27, 3.32) for the extreme-quartile comparison in the fully-adjusted model (*P*-trend =0.001; Additional file 1: Table S9). Each SD increment of the lipid score was associated with a 1.31-fold increased risk of GDM (95% CI: 1.09, 1.56; Fig. 2 and Additional file 1: Table S9).

Individual lipid metabolites could mediate the association between HSI and GDM risk, with mediating proportions ranging from 12.9% (PC[O-36:2]) to 34.3% (TG 18:1/18:1/22:6) (Fig. 2). The positive mediating role of lipid metabolites came from LPC, PC(O), TG, and Cer classes. Up to 52.6% of the association between HSI and GDM risk was attributed to the indirect effect of the HSI-related lipid score (Fig. 2).

## Discussion

In this prospective cohort study, elevated liver enzymes (ALT, AST, GGT, and ALP) and HSI (a reliable biomarker for NALFD) in early pregnancy were associated with increased risks of GDM, even within a normal range. A total of 15 lipid metabolites mainly from LPC, TG, SM, and Cer were independently associated with HSI. The association between HSI and GDM was largely mediated by HSI-related lipid metabolites. These findings indicate that NAFLD might increase GDM risk by disturbing the lipid metabolism.

The current large prospective study provided comprehensive and consistent evidence for the associations between different liver enzymes and subsequent risk of GDM. The positive association between GGT and GDM in our work was supported by a recent meta-analysis among 25,451 participants, in which GGT but not ALT or AST before gestation or in early pregnancy was positively associated with incident GDM [22]. However, consistent with our work, elevated ALT in early pregnancy was associated with an increased risk of GDM in a prospective study among 17,359 Chinese pregnant women [23], although two case-control studies among US and Chinese pregnant women showed no associations [24, 25]. Differences in the study design, diagnostic criteria for GDM, population characteristics (e.g., older age, higher BMI, and more frequent alcohol use during pregnancy), and limited sample sizes may partly explain the discrepant results for ALT. No previous studies examined the relation of AST in early pregnancy with risk of GDM, while other studies on pre-pregnancy AST showed no significant associations [35, 36]. Interestingly, restricted cubic spline analyses in our study showed nonlinear associations of ALT and AST with risk of GDM and the associations appeared to reach a plateau as ALT and AST elevated. Only one study that investigated the association between ALP and GDM found a linear association among 2,073 Chinese pregnant women [37], which is consistent with our findings. Intriguingly, pregnant women with elevated liver enzymes were at an increased risk of GDM even when the levels did not reach the threshold for clinical abnormalities in our work, which was reflected in other studies for ALT [23], GGT [24], and ALP [37]. While this finding should be confirmed elsewhere, it may suggest the need to routinely monitor the increase in liver enzymes in pregnant women.

NAFLD is a potential risk factor for insulin resistance. Although liver biopsy is the gold standard for diagnosing NAFLD, it is not feasible among asymptomatic pregnant women, and liver ultrasound could be an important alternative tool. Two small prospective cohort studies (*n*=476 and 608) showed that NAFLD based on liver ultrasound in early pregnancy predicted dysglycemia or GDM in mid pregnancy, and the risk of GDM increased up to 2.2-fold and 3.28-fold, respectively [4, 5]. However, liver ultrasound was reported to be a weak method for diagnosing NAFLD, especially when hepatic steatosis is mild [38, 39]. HSI can be applied as a noninvasive tool for screening NAFLD [6, 7], which is especially acceptable in pregnant women and reliable in the Asian population [7]. In the current study, elevated HSI was significantly associated with an increased risk of GDM in a dose-response manner, which agreed well with the finding from a recent cohort study using HSI [8]. Recent machine learning models also demonstrated that NAFLD-associated markers, especially HSI, significantly improved the predictive performance for GDM in early pregnancy [40]. When women with a normal liver ultrasound were included for analyses, an abnormal fatty liver index was still positively associated with an increased risk of GDM [5], which suggests that a noninvasive fatty liver scoring index may be useful as a predictive index in pregnant women with mild or moderate NAFLD. Collectively, our study with others indicated that HSI could be an alternative for identifying pregnant women at a higher risk for GDM. While our findings should be confirmed in future studies, the adoption of validated noninvasive hepatic markers into clinical practice could potentially be significant for GDM prevention and control.

Given that the primary driver of NAFLD is the expansion of adipose depots as well as the accumulation of ectopic fat [3], NAFLD may promote the remodeling of the fat distribution [41] and disturb the lipid metabolism, particularly in phospholipids (e.g., LPC, SM, and Cer) and glycerolipids (e.g., diacylglyceride and TG) [10–17]. Consistently, our work showed that 15 lipid metabolites mainly from LPC, TG, SM, and Cer were associated with HSI in pregnant women. In addition, we found that maternal phospholipids and glycerolipids (e.g., LPC 16:1 SN2, LPC 16:0, LPC 20:3, LPC 26:0, TG 18:1/18:1/22:6, and Cer 14:0) could mediate the association between HSI in early pregnancy and increased risk of GDM, and the HSI-related lipid metabolites explained approximately 52.6% of the association. These lend support to the hypothesis that HSI may increase GDM risk predominantly through disturbing the lipid metabolism. Of note, HSI was also significantly associated with systolic blood pressure, CRP, HOMA-IR, and clinical lipid biomarkers in our work. Our findings also suggest that mechanisms other than lipid metabolism should be examined in the future, such as chronic inflammation and blood pressure.

Current diagnosis for GDM is usually conducted at 24-28 weeks of gestation, which leaves a short time window for interventions. Thus, identifying potential risk factors for GDM is substantial for risk stratification. Since liver function test is a routine test in clinical practice during early pregnancy in China and these liver biomarkers are routinely examined as proxies for liver injury and fatty liver indicators, our findings highlight that early-pregnancy liver enzymes and HSI may be used for GDM risk stratification. Our identified lipid biomarkers for liver dysfunction in GDM development also suggest a direction for mechanistic studies. Future research work is warranted to validate our findings in other populations and to better understand the exact pathways of lipid disorders in the pathogenesis of GDM.

Our study has several notable strengths including a prospective study design, moderate sample size, comprehensive lipidomic measurement, and objective and comprehensive assessments of live function biomarkers. However, some limitations should be recognized. First, there is a lack of data for the utility of HSI in assessing liver dysfunctions among Chinese pregnant women, although HSI showed reasonable performance for screening NAFLD [6, 7] and was widely adopted as a biochemical indicator to identify the presence of NAFLD in many studies [42, 43]. Second, single baseline assessments may introduce measurement errors and do not capture the dynamics of liver biomarkers, so future studies are needed to understand how the dynamic changes of liver function biomarkers during pregnancy relate to GDM. Last, mediation analyses for the association between HSI and GDM were conducted in a subset of the study population with a limited sample size, so the findings should be validated in large prospective studies.

## Conclusions

In conclusion, elevated liver enzymes, and HSI as a potential biomarker for NAFLD in early pregnancy were associated with increased risks of GDM, even within a normal range in a Chinese birth cohort. Our findings also suggest that early-pregnancy HSI may increase GDM risk by altering lipid metabolism. Large prospective studies are warranted to confirm our findings in different populations and to investigate the biological mechanisms for the observed associations.

## Supplementary Information

**Additional file 1: Table S1.** Associations of liver biomarkers with blood glucose values in OGTT. **Table S2.** Joint associations of higher liver enzymes with risk of GDM. **Table S3.** Association of HSI with risk of GDM stratified by pre-pregnancy BMI and alcohol drinking status. **Table S4.** Associations of liver biomarkers with GDM after excluding participants with a history of GDM, current smokers, or current drinkers. **Table S5.** Associations of liver biomarkers with GDM among participants within relatively normal range of liver biomarkers. **Table S6.** Associations of liver biomarkers with GDM after multiple imputation. **Table S7.** Associations of liver biomarkers with GDM in Poisson regression. **Table S8.** Associations of liver biomarkers with GDM in a subset of 921 participants. **Table S9.** Association of HSI-related lipid score with GDM in a subset of 948 participants. **Figure S1.** Flowchart of the participant selection. **Figure S2.** Restricted cubic spline analyses for the associations of liver biomarkers with GDM risk. **Figure S3.** Spearman partial correlations between liver biomarkers and metabolic profiles.

## Data Availability

The datasets generated during and/or analyzed during the current study are available from the corresponding authors (pxiongfei@gmail.com or panan@hust.edu.cn) upon reasonable request.

## Abbreviations

ALP: Alkaline phosphatase
ALT: Alanine transaminase
AST: Aspartate aminotransferase
BMI: Body mass index
Cer: Ceramide
CI: Confidence interval
CRP: C-reactive protein
FDR: False discovery rate
FLI: Fatty liver index
GDM: Gestational diabetes mellitus
GGT: Gamma-glutamyltransferase
HDL-C: High-density lipoprotein cholesterol
HSI: Hepatic steatosis index
HOMA-IR: Homeostasis model assessment of insulin resistance
IQR: Interquartile range
LDL-C: Low-density lipoprotein cholesterol
LPC: Lysophosphatidylcholine
LPC(O): Lyso alkylphosphatidylcholine
MICE: Multiple imputations by chained equations
NAFLD: Non-alcoholic fatty liver disease
OGTT: Oral glucose tolerance test
OR: Odds ratio
PC: Phosphatidylcholine
PC(O): Alkylphosphatidylcholine
PC(P): Phosphatidylcholine plasmalogen
SD: Standard deviation
SM: Sphingomyelin
TG: Triacylglycerol
