# Supplementary material for: Liver biomarkers, lipid metabolites, and risk of gestational diabetes mellitus in a prospective study among Chinese pregnant women
Source: BMC Med. 2023 Apr 17;21:150. doi: 10.1186/s12916-023-02818-6 (PMC10111672; doi:10.1186/s12916-023-02818-6)
Supplement: Supplementary file 1 — Additional file 1: Table S1. Associations of liver biomarkers with blood glucose values in OGTT. Table S2. Joint associations of higher liver enzymes with risk of GDM. Table S3. Association of HSI with risk of GDM stratified by pre-pregnancy BMI and alcohol drinking status. Table S4. Associations of liver biomarkers with GDM after excluding participants with a history of GDM, current smokers, or current drinkers. Table S5. Associations of liver biomarkers with GDM among participants within relatively normal range of liver biomarkers. Table S6. Associations of liver biomarkers with GDM after multiple imputation. Table S7. Associations of liver biomarkers with GDM in Poisson regression. Table S8. Associations of liver biomarkers with GDM in a subset of 921 participants. Table S9. Association of HSI-related lipid score with GDM in a subset of 948 participants. Figure S1. Flowchart of the participant selection. Figure S2. Restricted cubic spline analyses for the associations of liver biomarkers with GDM risk. Figure S3. Spearman partial correlations between liver biomarkers and metabolic profiles. [file 12916_2023_2818_MOESM1_ESM.docx]

**Additional file 1**

**Liver biomarkers, lipid metabolites, and risk of gestational diabetes mellitus in a prospective study among Chinese pregnant women**

Ping Wu, Yi Wang, Yi Ye, Xue Yang, Yichao Huang, Yixiang Ye, Yuwei Lai, Jing Ouyang, Linjing Wu, Jianguo Xu, Jiaying Yuan, Yayi Hu, Yi-Xin Wang, Gang Liu, Da Chen, An Pan, Xiong-Fei Pan

**Table S1.** Associations of liver biomarkers with blood glucose values in OGTT.

**Table S2.** Joint associations of higher liver enzymes with risk of GDM.

**Table S3.** Association of HSI with risk of GDM stratified by pre-pregnancy BMI and alcohol drinking status.

**Table S4.** Odds ratios and 95% confidence intervals for the associations of liver biomarkers with GDM after excluding participants with a history of GDM, current smokers, or current drinkers.

**Table S5.** Odds ratios and 95% confidence intervals for the associations of liver biomarkers with GDM among participants within relatively normal range of liver biomarkers.

**Table S6.** Odds ratios and 95% confidence intervals for the associations of liver biomarkers with GDM after multiple imputation.

**Table S7.** Risk ratios and 95% confidence intervals for the associations of liver biomarkers with GDM in Poisson regression.

**Table S8.** Odds ratios and 95% confidence intervals for the associations of liver biomarkers with GDM in a subset of 921 participants.

**Table S9.** Odds ratios and 95% confidence intervals for the association of HSI-related lipid score with GDM in a subset of 948 participants.

**Figure S1.** Flowchart of the participant selection.

**Figure S2.** Restricted cubic spline analyses for the associations of liver biomarkers with GDM risk.

**Figure S3.** Spearman partial correlations between liver biomarkers and metabolic profiles.

**Table S1.** Associations of liver biomarkers with blood glucose values in the 75-g OGTT ^a^.

| Variables | OGTT fasting glucose | | OGTT 1-hour glucose | | OGTT 2-hour glucose | |
| --- | --- | --- | --- | --- | --- | --- |
|  | β (95% CI) | *P* value ^b^ | β (95% CI) | *P* value ^b^ | β (95% CI) | *P* value ^b^ |
| ALT, U/L | 0.008 (0.005, 0.012) | <.001 | 0.037 (0.027, 0.047) | <.001 | 0.031 (0.022, 0.039) | <.001 |
| AST, U/L | 0.009 (0.002, 0.015) | 0.008 | 0.055 (0.036, 0.073) | <.001 | 0.038 (0.022, 0.055) | <.001 |
| GGT, U/L | 0.017 (0.013, 0.021) | <.001 | 0.042 (0.031, 0.054) | <.001 | 0.035 (0.025, 0.046) | <.001 |
| ALP, U/L | 0.014 (0.006, 0.022) | <.001 | 0.038 (0.016, 0.061) | <.001 | 0.056 (0.036, 0.076) | <.001 |
| HSI ^c^ | 0.069 (0.051, 0.087) | <.001 | 0.218 (0.167, 0.270) | <.001 | 0.175 (0.129, 0.221) | <.001 |

Models adjusted for maternal age, gestational age, parity, family history of diabetes, history of GDM, pre-pregnancy BMI, systolic blood pressure, smoking status, alcohol drinking status, physical activity, and fasting blood glucose. ^a^ Liver biomarkers, and blood glucose at OGTT test were natural log-transformed. Numbers of participants were 6585 for OGTT fasting glucose, 6538 for 1-hour glucose, and 6536 for 2-hour glucose. ^b^ *P* values were false discovery rate corrected. ^c^ Pre-pregnancy BMI was treated as a categorical variable (<18.5, 18.5-24.0, and ≥24.0 kg/m^2^) in the model.

Abbreviations: ALP, alkaline phosphatase; ALT, alanine transaminase; AST, aspartate aminotransferase; BMI, body mass index; CI, confidence interval; GDM, gestational diabetes mellitus; GGT, gamma-glutamyltransferase; HSI, hepatic steatosis index; OGTT, oral glucose tolerance test.

**Table S2.** Joint associations of higher liver enzymes with risk of GDM.

| Joint effect of four liver enzymes ^a^ | Case/total (%) | OR (95% CI) | |
| --- | --- | --- | --- |
|  |  | Crude model | Multivariable model ^b^ |
| 0 | 65/1385 (4.69) | 1.00 (reference) | 1.00 (reference) |
| 1 | 84/1532 (5.48) | 1.18 (0.85, 1.64) | 1.13 (0.80, 1.60) |
| 2 | 90/1371 (6.56) | 1.43 (1.03, 1.98) | 1.39 (0.99, 1.96) |
| 3 | 110/1300 (8.46) | 1.88 (1.37, 2.58) | 1.63 (1.17, 2.27) |
| 4 | 143/1272 (11.24) | 2.57 (1.90, 3.49) | 1.95 (1.41, 2.69) |

^a^ Four liver enzymes were dichotomized into two groups by their median values. ^b^ Multivariable model was adjusted for maternal age, gestational age, parity, family history of diabetes, history of GDM, pre-pregnancy BMI, systolic blood pressure, smoking status, alcohol drinking status, physical activity, and fasting blood glucose.

Abbreviations: BMI, body mass index; CI, confidence interval; GDM, gestational diabetes mellitus; OR, odds ratio.

**Table S3** Association of HSI with risk of GDM stratified by pre-pregnancy BMI and alcohol drinking status.

| Variables | Case/total (%) | Median (IQR) | OR (95% CI) ^a^ | *P* for interaction |
| --- | --- | --- | --- | --- |
| Pre-pregnancy BMI, kg/m^2^ |  |  |  | 0.74 |
| <24.0 | 363/5898 (6.2) | 29.3 (27.1-31.8) | 1.36 (1.19, 1.55) |  |
| ≥24.0 | 129/962 (13.4) | 37.2 (34.8-40.5) | 1.20 (0.93, 1.56) |  |
| Alcohol drinking |  |  |  | 0.17 |
| Never | 386/5443 (7.1) | 30.0 (27.4-33.5) | 1.29 (1.12, 1.49) |  |
| Current or former | 106/1417 (7.5) | 30.1 (27.6-33.5) | 1.46 (1.13, 1.89) |  |

^a^ HSI was modelled as SD increment on the log scale. Model was adjusted for maternal age, gestational age, parity, family history of diabetes, history of GDM, pre-pregnancy BMI, systolic blood pressure, smoking status, alcohol drinking status, physical activity, and fasting blood glucose except for stratification factors. Pre-pregnancy BMI was treated as a categorical variable (<18.5, 18.5-24.0, and ≥24.0 kg/m^2^) for adjustment in the modeling to avoid multicollinearity.

Abbreviations: BMI, body mass index; CI, confidence interval; GDM, gestational diabetes mellitus; HSI, hepatic steatosis index; IQR, interquartile range; OR, odds ratio; SD, standard deviation.

**Table S4.** Odds ratios and 95% confidence intervals for the associations of liver biomarkers with GDM after excluding participants with a history of GDM, current smokers, or current drinkers.

| Variables | Quartiles of liver biomarkers | | | | *P*-trend ^a^ | Per SD increment on the log scale | *P* for SD increment analysis ^a^ |
| --- | --- | --- | --- | --- | --- | --- | --- |
|  | Quartile 1 | Quartile 2 | Quartile 3 | Quartile 4 |  |  |  |
| ALT | 1.00 (reference) | 1.34 (0.97, 1.84) | 1.62 (1.21, 2.18) | 1.81 (1.35, 2.42) | <.001 | 1.16 (1.06, 1.28) | 0.004 |
| AST | 1.00 (reference) | 1.41 (1.05, 1.90) | 1.51 (1.15, 2.00) | 1.54 (1.17, 2.03) | 0.007 | 1.10 (1.00, 1.21) | 0.04 |
| GGT | 1.00 (reference) | 0.79 (0.58, 1.09) | 1.15 (0.86, 1.54) | 1.43 (1.07, 1.90) | <.001 | 1.18 (1.08, 1.30) | 0.001 |
| ALP | 1.00 (reference) | 0.93 (0.69, 1.26) | 1.18 (0.89, 1.56) | 1.40 (1.06, 1.85) | 0.007 | 1.16 (1.05, 1.28) | 0.005 |
| HSI ^b^ | 1.00 (reference) | 1.36 (0.94, 1.96) | 1.65 (1.14, 2.39) | 2.38 (1.62, 3.51) | <.001 | 1.35 (1.18, 1.53) | <.001 |

Model adjusted for maternal age, gestational age, parity, family history of diabetes, history of GDM, pre-pregnancy BMI, systolic blood pressure, smoking status, alcohol drinking status, physical activity, and fasting blood glucose after excluding participants with a history of GDM, current smokers, or current drinkers (n=6643). ^a^ *P* values were false discovery rate corrected. ^b^ Pre-pregnancy BMI was treated as a categorical variable (<18.5, 18.5-24.0, and ≥24.0 kg/m^2^) for adjustment in the modeling to avoid multicollinearity.

Abbreviations: ALP, alkaline phosphatase; ALT, alanine transaminase; AST, aspartate aminotransferase; BMI, body mass index; GDM, gestational diabetes mellitus; GGT, gamma-glutamyltransferase; HSI, hepatic steatosis index; SD, standard deviation.

**Table S5.** Odds ratios and 95% confidence intervals for the associations of liver biomarkers with GDM among participants within relatively normal range of liver biomarkers.

| Variables | Quartiles of liver biomarkers | | | | *P*-trend ^a^ | Per SD increment on the log scale | *P* for SD increment analysis ^a^ |
| --- | --- | --- | --- | --- | --- | --- | --- |
|  | Quartile 1 | Quartile 2 | Quartile 3 | Quartile 4 |  |  |  |
| ALT (n=6282) | 1.00 (reference) | 1.22 (0.88, 1.69) | 1.53 (1.12, 2.09) | 1.65 (1.21, 2.25) | 0.001 | 1.23 (1.11, 1.37) | <.001 |
| AST (n=6691) | 1.00 (reference) | 1.46 (1.10, 1.93) | 1.50 (1.13, 1.99) | 1.57 (1.21, 2.04) | 0.002 | 1.15 (1.04, 1.26) | 0.004 |
| GGT (n=6704) | 1.00 (reference) | 0.85 (0.63, 1.15) | 1.29 (0.97, 1.72) | 1.46 (1.10, 1.94) | <.001 | 1.24 (1.13, 1.37) | <.001 |
| ALP (n=5225) | 1.00 (reference) | 1.09 (0.79, 1.51) | 1.26 (0.91, 1.73) | 1.67 (1.24, 2.24) | <.001 | 1.20 (1.08, 1.32) | <.001 |
| HSI (n=5908) ^b^ | 1.00 (reference) | 1.55 (1.06, 2.27) | 1.56 (1.05, 2.31) | 2.20 (1.48, 3.27) | <.001 | 1.38 (1.20, 1.58) | <.001 |
| Completely normal liver enzymes (n=4637) ^c^ | | | | | | | |
| ALT | 1.00 (reference) | 1.06 (0.73, 1.53) | 1.57 (1.11, 2.21) | 1.56 (1.11, 2.20) | 0.004 | 1.23 (1.09, 1.39) | 0.002 |
| AST | 1.00 (reference) | 1.43 (1.03, 2.00) | 1.51 (1.08, 2.11) | 1.53 (1.10, 2.12) | 0.02 | 1.16 (1.03, 1.30) | 0.02 |
| GGT | 1.00 (reference) | 1.10 (0.75, 1.62) | 1.40 (1.01, 1.94) | 1.64 (1.19, 2.26) | 0.003 | 1.26 (1.13, 1.41) | <.001 |
| ALP | 1.00 (reference) | 1.21 (0.85, 1.73) | 1.36 (0.96, 1.94) | 1.70 (1.21, 2.38) | 0.003 | 1.19 (1.06, 1.33) | 0.003 |
| HSI ^b^ | 1.00 (reference) | 1.26 (0.81, 1.94) | 1.32 (0.85, 2.06) | 2.17 (1.36, 3.45) | 0.002 | 1.39 (1.18, 1.63) | <.001 |
| HSI ≤36 (n=5908) | | | | | | | |
| ALT | 1.00 (reference) | 1.15 (0.82, 1.60) | 1.61 (1.17, 2.22) | 1.65 (1.20, 2.28) | 0.002 | 1.22 (1.10, 1.36) | <.001 |
| AST | 1.00 (reference) | 1.25 (0.92 1.70) | 1.41 (1.01, 1.98) | 1.67 (1.21, 2.30) | 0.002 | 1.15 (1.04, 1.28) | 0.009 |
| GGT | 1.00 (reference) | 0.85 (0.62, 1.16) | 1.17 (0.85, 1.62) | 1.52 (1.14, 2.05) | 0.001 | 1.23 (1.12, 1.36) | <.001 |
| ALP | 1.00 (reference) | 0.87 (0.63, 1.19) | 1.17 (0.85, 1.59) | 1.30 (1.96, 1.77) | 0.03 | 1.15 (1.03, 1.28) | 0.01 |

Model was adjusted for maternal age, gestational age, parity, family history of diabetes, history of GDM, pre-pregnancy BMI, systolic blood pressure, smoking status, alcohol drinking status, physical activity, and fasting blood glucose. The relatively healthy range of liver biomarkers: ALT ≤40 U/L, AST ≤40 U/L, GGT ≤50 U/L, ALP range from 40 U/L to 150 U/L, and HSI ≤36. ^a^ *P* values were false discovery rate corrected. ^b^ Pre-pregnancy BMI was treated as a categorical variable (<18.5, 18.5-24.0, and ≥24.0 kg/m^2^) for adjustment in the modeling to avoid multicollinearity. ^c^ Completely normal liver enzymes were defined as ALT ≤40 U/L, AST ≤40 U/L, GGT ≤ 50 U/L, and ALP from 40 to 150 U/L.

Abbreviations: ALP, alkaline phosphatase; ALT, alanine transaminase; AST, aspartate aminotransferase; BMI, body mass index; GDM, gestational diabetes mellitus; GGT, gamma-glutamyltransferase; HSI, hepatic steatosis index; SD, standard deviation.

**Table S6.** Odds ratios and 95% confidence intervals for the associations of liver biomarkers with GDM after multiple imputation.

| Variables | Quartiles of liver biomarkers | | | | *P*-trend ^a^ | Per SD increment on the log scale | *P* for SD increment analysis ^a^ |
| --- | --- | --- | --- | --- | --- | --- | --- |
|  | Quartile 1 | Quartile 2 | Quartile 3 | Quartile 4 |  |  |  |
| ALT | 1.00 (reference) | 1.25 (0.92, 1.71) | 1.51 (1.14, 2.01) | 1.67 (1.26, 2.21) | <.001 | 1.14 (1.04, 1.25) | 0.009 |
| AST | 1.00 (reference) | 1.41 (1.07, 1.87) | 1.44 (1.11, 1.88) | 1.49 (1.15, 1.95) | 0.01 | 1.08 (0.99, 1.19) | 0.08 |
| GGT | 1.00 (reference) | 0.85 (0.63, 1.16) | 1.26 (0.95, 1.67) | 1.53 (1.15, 2.04) | <.001 | 1.21 (1.10, 1.32) | <.001 |
| ALP | 1.00 (reference) | 0.99 (0.74, 1.33) | 1.22 (0.93, 1.60) | 1.42 (1.08, 1.86) | 0.005 | 1.14 (1.04, 1.26) | 0.009 |
| HSI ^b^ | 1.00 (reference) | 1.30 (0.91, 1.84) | 1.64 (1.16, 2.34) | 2.24 (1.55, 3.26) | <.001 | 1.33 (1.17, 1.51) | <.001 |

Model was adjusted for maternal age, gestational age, parity, family history of diabetes, history of GDM, pre-pregnancy BMI, systolic blood pressure, smoking status, alcohol drinking status, physical activity, and fasting blood glucose. ^a^ *P* values were false discovery rate corrected. ^b^ Pre-pregnancy BMI was treated as a categorical variable (<18.5, 18.5-24.0, and ≥24.0 kg/m^2^) for adjustment in the modeling to avoid multicollinearity. Missing data (exposure, covariates, and outcome) were imputed using multiple imputations by chained equations with 10 imputations (n=7269). The “multiple imputation, then deletion” approach was used, for which observations with imputed outcomes were excluded from the analysis (n=7013). The imputed data were incorporated into the final model using Rubin's rules.

Abbreviations: ALP, alkaline phosphatase; ALT, alanine transaminase; AST, aspartate aminotransferase; BMI, body mass index; GDM, gestational diabetes mellitus; GGT, gamma-glutamyltransferase; HSI, hepatic steatosis index; SD, standard deviation.

**Table S7.** Risk ratios and 95% confidence intervals for the associations of liver biomarkers with GDM in Poisson regression.

| Variables |  | Quartiles of liver biomarkers | | | *P*-trend ^a^ | Per SD increment on the log scale | *P* for SD increment analysis ^a^ |
| --- | --- | --- | --- | --- | --- | --- | --- |
|  | Quartile 1 | Quartile 2 | Quartile 3 | Quartile 4 |  |  |  |
| ALT, U/L |  |  |  |  |  |  |  |
| Crude model | 1.00 (reference) | 1.39 (1.05, 1.83) | 1.75 (1.35, 2.26) | 2.25 (1.76, 2.87) | <.001 | 1.27 (1.18, 1.36) | <.001 |
| Multivariable model | 1.00 (reference) | 1.23 (0.94, 1.62) | 1.44 (1.12, 1.86) | 1.61 (1.25, 2.06) | <.001 | 1.14 (1.05, 1.22) | 0.002 |
| AST, U/L |  |  |  |  |  |  |  |
| Crude model | 1.00 (reference) | 1.36 (1.06, 1.75) | 1.46 (1.15, 1.85) | 1.68 (1.33, 2.13) | <.001 | 1.15 (1.07, 1.23) | <.001 |
| Multivariable model | 1.00 (reference) | 1.40 (1.10, 1.78) | 1.42 (1.13, 1.78) | 1.47 (1.17, 1.85) | 0.005 | 1.09 (1.01, 1.17) | 0.02 |
| GGT, U/L |  |  |  |  |  |  |  |
| Crude model | 1.00 (reference) | 0.96 (0.72, 1.26) | 1.41 (1.09, 1.81) | 2.03 (1.59, 2.58) | <.001 | 1.35 (1.26, 1.44) | <.001 |
| Multivariable model | 1.00 (reference) | 0.87 (0.66, 1.14) | 1.22 (0.96, 1.57) | 1.42 (1.11, 1.81) | <.001 | 1.15 (1.07, 1.24) | <.001 |
| ALP, U/L |  |  |  |  |  |  |  |
| Crude model | 1.00 (reference) | 1.03 (0.79, 1.35) | 1.30 (1.02, 1.66) | 1.59 (1.26, 2.02) | <.001 | 1.20 (1.10, 1.31) | <.001 |
| Multivariable model | 1.00 (reference) | 0.98 (0.76, 1.27) | 1.18 (0.93, 1.50) | 1.32 (1.05, 1.68) | 0.007 | 1.12 (1.02, 1.22) | 0.02 |
| HSI ^b^ |  |  |  |  |  |  |  |
| Crude model | 1.00 (reference) | 1.42 (1.05, 1.94) | 1.88 (1.40, 2.51) | 3.15 (2.41, 4.12) | <.001 | 1.48 (1.38, 1.58) | <.001 |
| Multivariable model | 1.00 (reference) | 1.29 (0.93, 1.78) | 1.57 (1.13, 2.18) | 2.10 (1.50, 2.94) | <.001 | 1.30 (1.17, 1.43) | <.001 |

Multivariable model was adjusted for maternal age, gestational age, parity, family history of diabetes, history of GDM, pre-pregnancy BMI, systolic blood pressure, smoking status, alcohol drinking status, physical activity, and fasting blood glucose. ^a^ *P* values were false discovery rate corrected for each stepwise modeling. ^b^ Pre-pregnancy BMI was treated as a categorical variable (<18.5, 18.5-24.0, and ≥24.0 kg/m^2^) for adjustment in the modeling to avoid multicollinearity.

Abbreviations: ALP, alkaline phosphatase; ALT, alanine transaminase; AST, aspartate aminotransferase; BMI, body mass index; GDM, gestational diabetes mellitus; GGT, gamma-glutamyltransferase; HSI, hepatic steatosis index; SD, standard deviation.

**Table S8.** Odds ratios and 95% confidence intervals for the associations of liver biomarkers with GDM in a subset of 921 participants.

| Variables | Quartiles of liver biomarkers | | | | Per SD increment on log scale |
| --- | --- | --- | --- | --- | --- |
|  | Quartile 1 | Quartile 2 | Quartile 3 | Quartile 4 |  |
| ALT |  |  |  |  |  |
| Model 1 | 1.00 (reference) | 1.49 (0.94, 2.36) | 1.69 (1.06, 2.71) | 1.95 (1.21, 3.15) | 1.20 (1.02, 1.41) |
| Model 2 | 1.00 (reference) | 1.48 (0.93, 2.37) | 1.71 (1.06, 2.77) | 1.93 (1.18, 3.15) | 1.19 (1.01, 1.41) |
| Model 3 | 1.00 (reference) | 1.51 (0.94, 2.42) | 1.70 (1.05, 2.76) | 1.93 (1.18, 3.18) | 1.19 (1.01, 1.41) |
| AST |  |  |  |  |  |
| Model 1 | 1.00 (reference) | 1.65 (1.05, 2.59) | 1.34 (0.87, 2.06) | 1.97 (1.26, 3.08) | 1.16 (1.00, 1.35) |
| Model 2 | 1.00 (reference) | 1.63 (1.03, 2.56) | 1.32 (0.85, 2.05) | 1.89 (1.20, 2.98) | 1.15 (0.99, 1.34) |
| Model 3 | 1.00 (reference) | 1.67 (1.06, 2.64) | 1.35 (0.87, 2.09) | 1.94 (1.23, 3.07) | 1.16 (0.99, 1.35) |
| GGT |  |  |  |  |  |
| Model 1 | 1.00 (reference) | 1.10 (0.71, 1.71) | 1.23 (0.80, 1.87) | 1.26 (0.84, 1.88) | 1.08 (0.92, 1.25) |
| Model 2 | 1.00 (reference) | 1.10 (0.70, 1.71) | 1.27 (0.82, 1.96) | 1.19 (0.78, 1.80) | 1.05 (0.90, 1.23) |
| Model 3 | 1.00 (reference) | 1.09 (0.70, 1.71) | 1.27 (0.83, 1.97) | 1.17 (0.77, 1.77) | 1.04 (0.89, 1.22) |
| ALP |  |  |  |  |  |
| Model 1 | 1.00 (reference) | 1.09 (0.71, 1.67) | 1.05 (0.68, 1.61) | 1.40 (0.91, 2.15) | 1.07 (0.91, 1.25) |
| Model 2 | 1.00 (reference) | 1.01 (0.65, 1.56) | 0.97 (0.63, 1.51) | 1.32 (0.85, 2.04) | 1.04 (0.89, 1.22) |
| Model 3 | 1.00 (reference) | 0.99 (0.64, 1.53) | 0.96 (0.62, 1.49) | 1.28 (0.82, 1.99) | 1.03 (0.88, 1.21) |
| HSI ^a^ |  |  |  |  |  |
| Model 1 | 1.00 (reference) | 1.56 (0.98, 2.48) | 1.70 (1.07, 2.72) | 2.27 (1.42, 3.61) | 1.30 (1.11, 1.53) |
| Model 2 | 1.00 (reference) | 1.48 (0.92, 2.38) | 1.62 (1.00, 2.62) | 2.10 (1.29, 3.42) | 1.28 (1.08, 1.51) |
| Model 3 | 1.00 (reference) | 1.47 (0.91, 2.37) | 1.56 (0.96, 2.54) | 1.90 (1.15, 3.14) | 1.23 (1.04, 1.47) |
| FLI ^b^ |  |  |  |  |  |
| Model 1 | 1.00 (reference) | 1.10 (0.70, 1.74) | 1.19 (0.76, 1.86) | 1.69 (1.08, 2.63) | 1.28 (1.09, 1.50) |
| Model 2 | 1.00 (reference) | 1.07 (0.67, 1.71) | 1.08 (0.67, 1.72) | 1.50 (0.94, 2.39) | 1.23 (1.04, 1.46) |
| Model 3 | 1.00 (reference) | 1.04 (0.65, 1.65) | 1.00 (0.62, 1.60) | 1.30 (0.80, 2.12) | 1.17 (0.97, 1.40) |

Model 1 was adjusted for maternal age, gestational age, parity, family history of diabetes, history of GDM, pre-pregnancy BMI, systolic blood pressure, smoking status, alcohol drinking status, physical activity, and fasting blood glucose. Model 2 was additionally adjusted for CRP, total cholesterol, and triglycerides based on Model 1. Model 3 was additionally adjusted for HOMA-IR based on Model 2. ^a^ Pre-pregnancy BMI was excluded in the multivariable model. ^b^ FLI = exp(model) / (1+exp[model])×100, where model = (0.953 × log [triglycerides (mmol/L) × 88.5]) + (0.139 × BMI [kg/m^2^]) + (0.718 × log GGT [U/L]) + (0.053 × waist circumference [cm]) -15.745). Pre-pregnancy BMI and triglycerides were excluded from the multivariable model for FLI.

Abbreviations: ALP, alkaline phosphatase; ALT, alanine transaminase; AST, aspartate aminotransferase; BMI, body mass index; CRP, C-reactive protein; FLI, fatty liver index; GDM, gestational diabetes mellitus; GGT, gamma-glutamyltransferase; HOMA-IR, homeostasis model assessment of insulin resistance; HSI, hepatic steatosis index; SD, standard deviation.

**Table S9.** Odds ratios and 95% confidence intervals for the association of HSI-related lipid score with GDM in a subset of 948 participants.

|  | Quartiles of lipid score | | | | *P*-trend | Per SD increment | *P* for SD increment |
| --- | --- | --- | --- | --- | --- | --- | --- |
|  | Quartile 1 | Quartile 2 | Quartile 3 | Quartile 4 |  |  |  |
| Case/control | 47/158 | 60/158 | 89/158 | 120/158 |  |  |  |
| Crude model | 1.00 (reference) | 1.30 (0.83, 2.03) | 1.98 (1.30, 3.03) | 2.76 (1.80, 4.23) | <.001 | 1.49 (1.28, 1.73) | <.001 |
| Multivariable model | 1.00 (reference) | 1.09 (0.68, 1.75) | 1.54 (0.97, 2.44) | 2.05 (1.27, 3.32) | 0.001 | 1.31 (1.09, 1.56) | 0.003 |

Multivariable model was adjusted for maternal age, gestational age, parity, family history of diabetes, history of GDM, pre-pregnancy BMI, systolic blood pressure, smoking status, alcohol drinking status, physical activity, and fasting blood glucose.

Abbreviations: BMI, body mass index; GDM, gestational diabetes mellitus; HSI, hepatic steatosis index; SD, standard deviation.

**
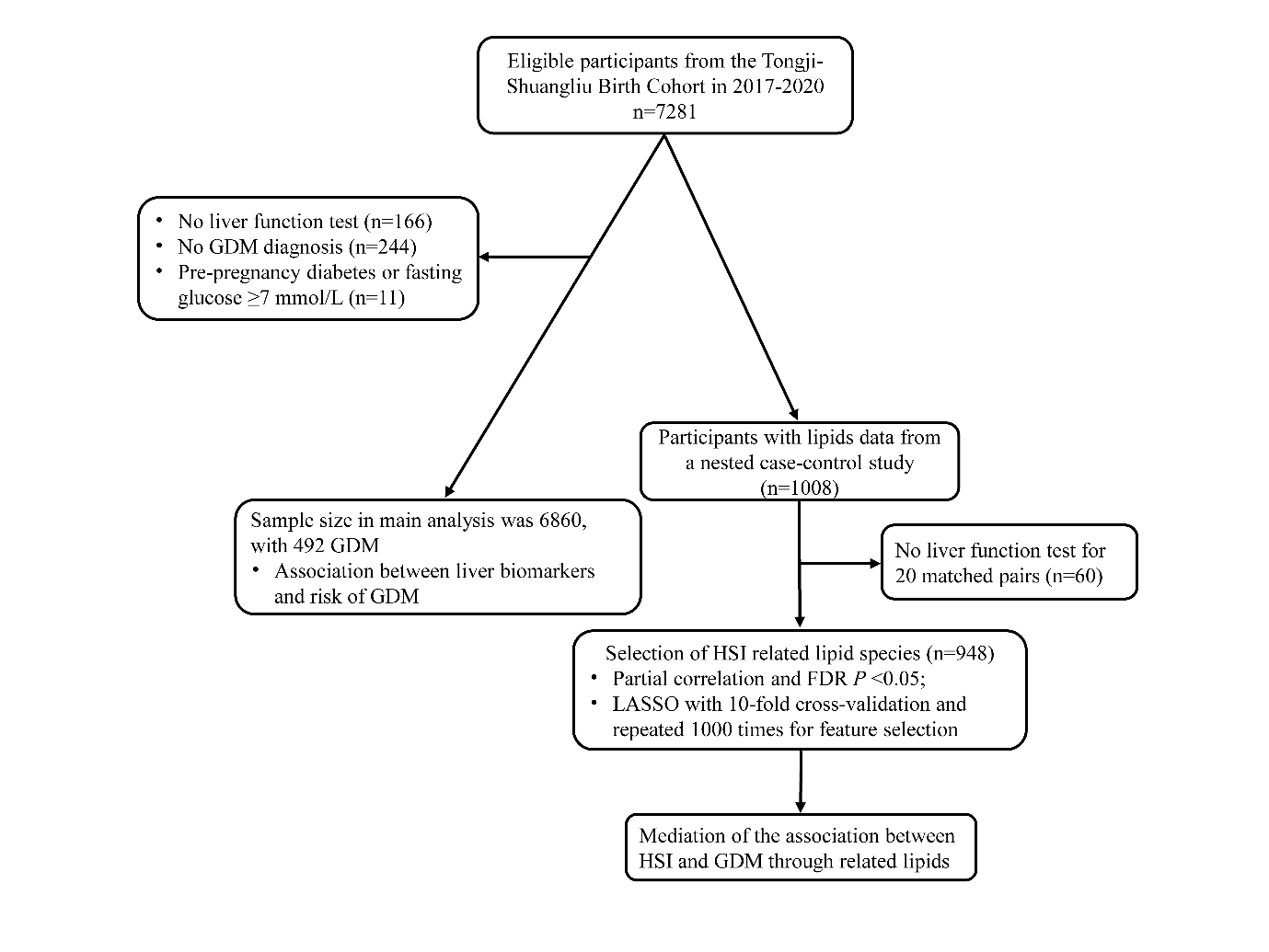
Figure S1.** Flowchart of the participant selection.

Abbreviations: FDR, false discovery rate; GDM, gestational diabetes mellitus; HSI, hepatic steatosis index; LASSO, least absolute shrinkage and selection operator.

**
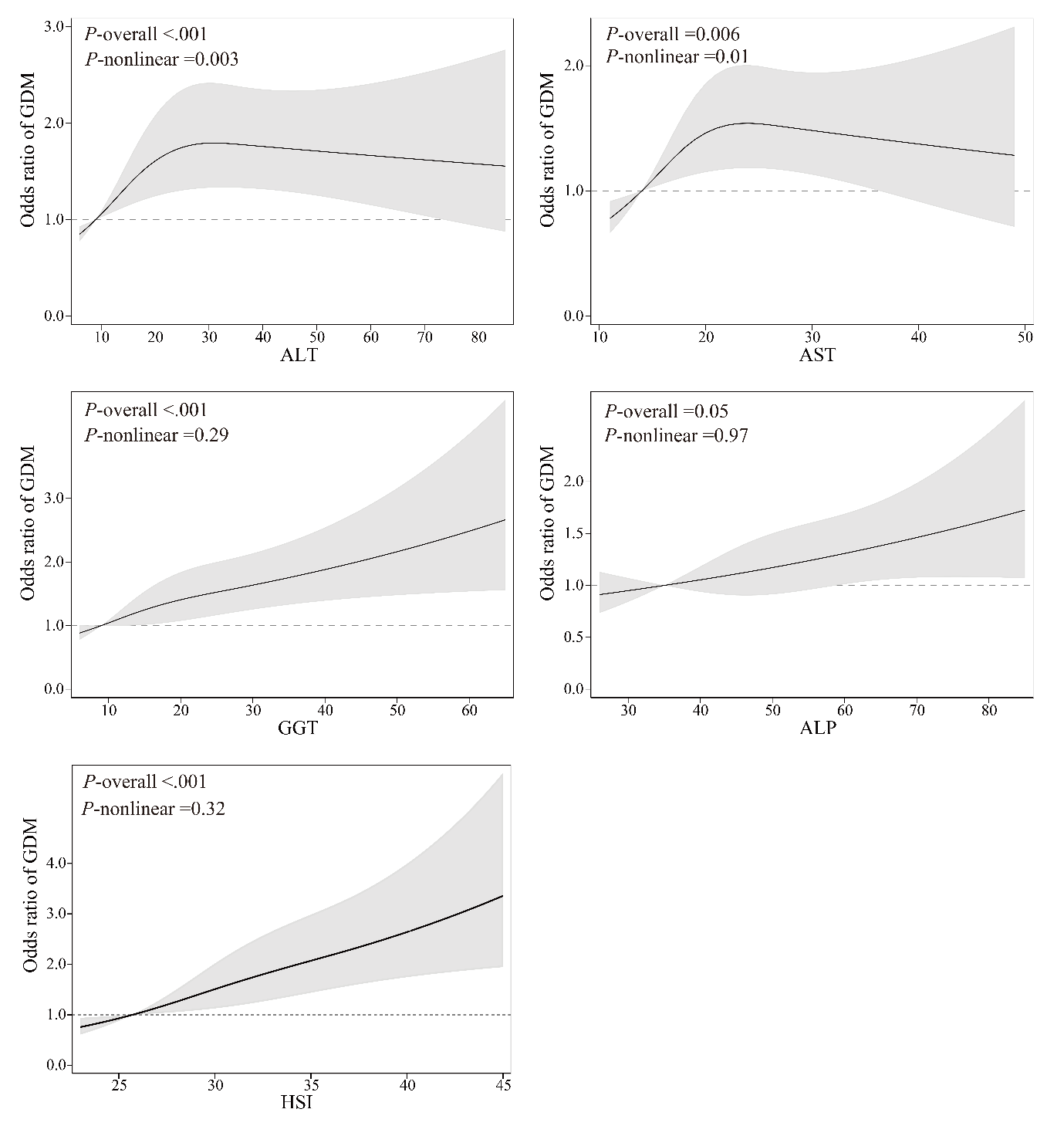
**

**Figure S2.** Restricted cubic spline analyses for the associations of liver biomarkers with GDM risk.

Models were adjusted for maternal age, gestational age, parity, family history of diabetes, history of GDM, pre-pregnancy BMI, systolic blood pressure, smoke status, alcohol drinking status, physical activity, and fasting blood glucose with 3 knots at 10 (reference), 50 and 90 percentiles of liver markers after excluding extreme values (<1% and >99% of liver biomarkers).

Abbreviations: ALP, alkaline phosphatase; ALT, alanine transaminase; AST, aspartate aminotransferase; BMI, body mass index; GDM, gestational diabetes mellitus; GGT, gamma-glutamyltransferase; HSI, hepatic steatosis index.

**
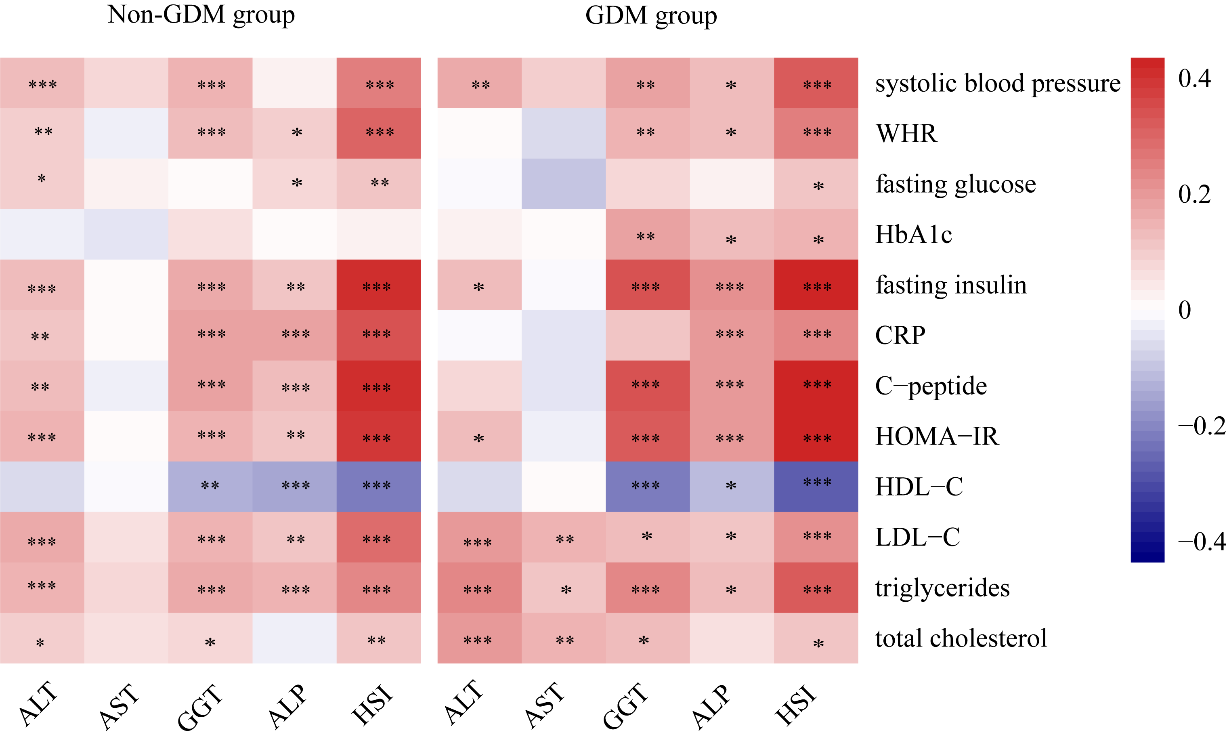
**

**Figure S3.** Spearman partial correlations between liver biomarkers and metabolic profiles.

Correlation coefficients were estimated after adjustment for maternal age and gestational age among participants with and without GDM (n=649 and 325, respectively). Among the 6860 participants in the main analysis, there were 974 participants with data on these metabolic biomarkers.

**P* <0.05, ***P* <0.01, ****P* <0.001

Abbreviations: ALP, alkaline phosphatase; ALT, alanine transaminase; AST, aspartate aminotransferase; BMI, body mass index; CRP, C-reactive protein; GDM, gestational diabetes mellitus; GGT, gamma-glutamyltransferase; HbA1c, hemoglobin A1c; HDL-C, high-density lipoprotein cholesterol; HSI, hepatic steatosis index; LDL-C, low-density lipoprotein cholesterol; WHR, waist-hip ratio.
